# Supplementary material for: Diastereoisomerically Pure, (S)-O-1,2-O-isopropyli dene-(5-O-α-d-glucofuranosyl) t-butanesulfinate: Synthesis, Crystal Structure, Absolute Configuration and Reactivity
Source: Molecules. 2020 Jul 27;25(15):3392. doi: 10.3390/molecules25153392 (PMC7436146; doi:10.3390/molecules25153392)
Supplement: Supplementary file 1 [file molecules-25-03392-s001.pdf]

Supporting Information

Diastereoisomerically pure, (*S*)-*O*-1,2-*O*-isopropylidene-(5-*O*-*α*-D-glucofuranosyl) *t*-butanesulfinate: Synthesis, crystal structure, absolute configuration and reactivity

Bogdan Bujnicki[†], Jarosław Błaszczyk[†,*], Marek Chmielewski[‡], and Józef Drabowicz[†,§,**]

[†]Centre of Molecular and Macromolecular Studies, Polish Academy of Sciences, Division of Organic Chemistry, Sienkiewicza 112, 90-363 Łódź, Poland.
[‡] Institute of Organic Chemistry, Polish Academy of Sciences, Kasprzaka 44/52, 01-224 Warszawa, Poland.
[§] Jan Długosz University, Institute of Chemistry, Aleja Armii Krajowej 13/15, 42-200 Częstochowa, Poland.

Table S1. Crystal data and experimental details for (*S*)-1,2-*O*-isopropylidene-(5-*O*-α-D-glucofuranosyl) *t*-butanesulfinate **10**.

Molecular formula	$C_{13}H_{24}O_7S$
Formula weight	324.4
Crystallographic system	Orthorhombic
Space group	$P2_12_12_1$
a [Å]	7.097(3)
b [Å]	9.594(3)
c [Å]	23.832(5)
V [$Å^3$]	1622.7(9)
Z	4
D_{calcd} [g/cm^3]	1.328
Data collection temperature [K]	100(2)
Crystal dimensions [mm]	0.3, 0.2, 0.1
F(000)	696
Radiation, λ [Å]	MoKα, 0.71073
Scan mode	ω
$2\Theta_{max}$ [°]	72.28
hkl ranges	h (-11,11), k (-15,9), l (-31,37)
Absorption coefficient μ [mm^{-1}]	0.228
Reflections measured	17405
Reflections unique	6250
Reflections observed, I > 2σ(I)	5270
R_{int}	0.0375
Parameters refined	263
Flack x parameter	-0.11(9)
wR^2 (obs)	0.1440
R_1(obs)	0.0633
S (goodness-of-fit)	1.067
Residual density max [$eÅ^{-3}$]	0.381
Residual density min [$eÅ^{-3}$]	-0.537
Resolution max [Å]	0.6026

Table S2. Asymmetry parameters for the two five-membered rings in the crystal structure of (*S*)-1,2-*O*-isopropylidene-(5-*O*-α-D-glucofuranose) *t*-butanesulfinate **10**.

Ring C1,C2,C3,C4,O4			
ΔC_s^{C}	47.5(4)	$\Delta C_2^{C1\text{-}C2}$	59.1(4)
ΔC_s^{C}	39.2(4)	$\Delta C_2^{C2\text{-}C3}$	38.9(4)
ΔC_s^{C}	17.4(4)	$\Delta C_2^{C3\text{-}C4}$	4.1(4)
ΔC_s^{C}	11.0(4)	$\Delta C_2^{C4\text{-}O4}$	32.7(4)
ΔC_s^{O}	37.0(4)	$\Delta C_2^{O4\text{-}C1}$	56.8(4)

Ring C1,C2,O2,C7,O1			
ΔC_s^{C1}	36.0(5)	$\Delta C_2^{C1\text{-}C2}$	40.9(6)
ΔC_s^{C2}	24.1(5)	$\Delta C_2^{C2\text{-}O2}$	17.5(6)
ΔC_s^{O2}	2.2(6)	$\Delta C_2^{O2\text{-}C7}$	12.6(6)
ΔC_s^{C7}	20.8(5)	$\Delta C_2^{C7\text{-}O1}$	37.9(6)
ΔC_s^{O1}	35.4(5)	$\Delta C_2^{O1\text{-}C1}$	48.7(6)

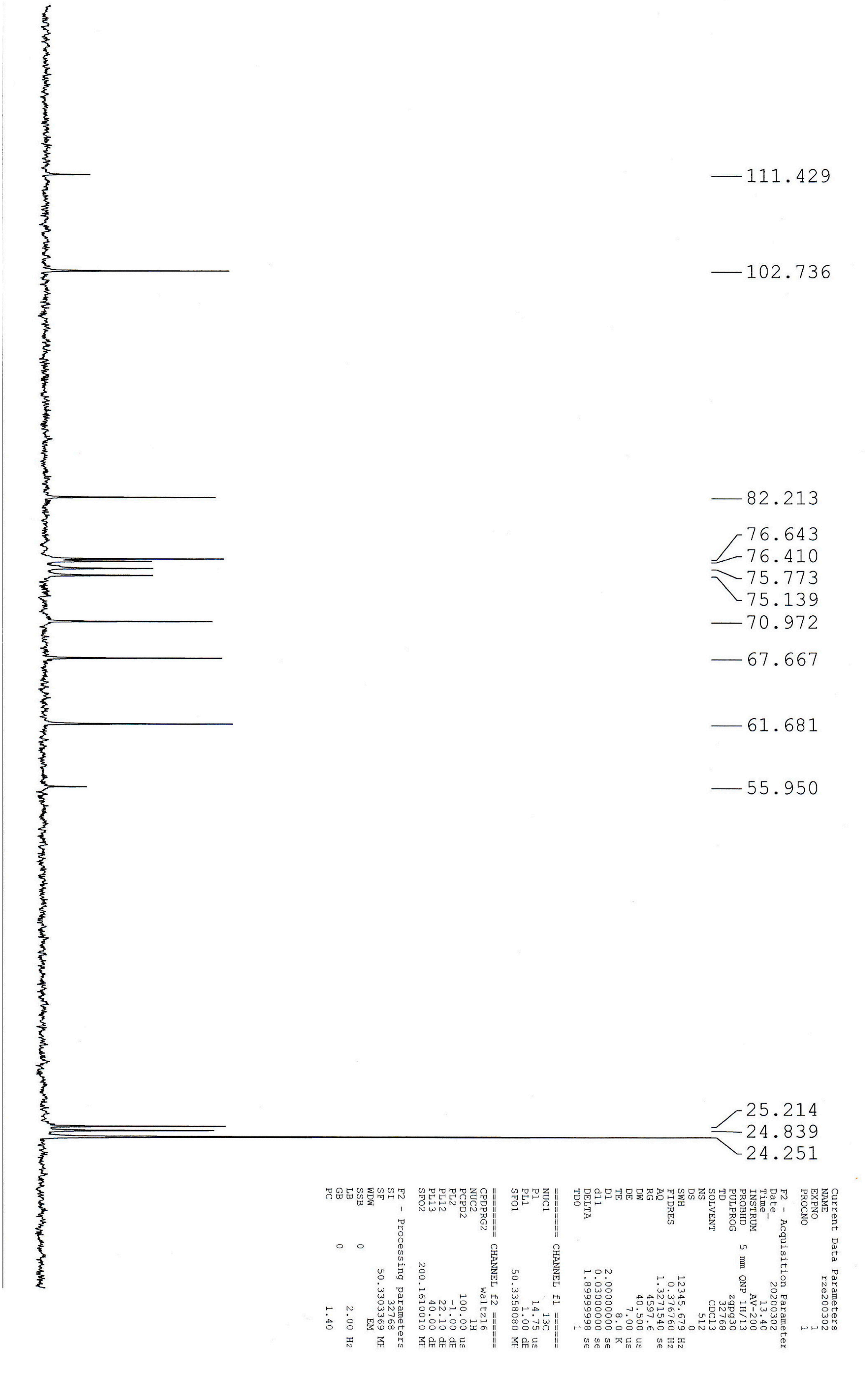

Figure 1S. ^{13}C NMR Spectrum of (*S*)-1,2-*O*-isopropylidene-(5-*O*-α-D-glucofuranosyl) *t*-butanesulfinate (*S*)-**10**

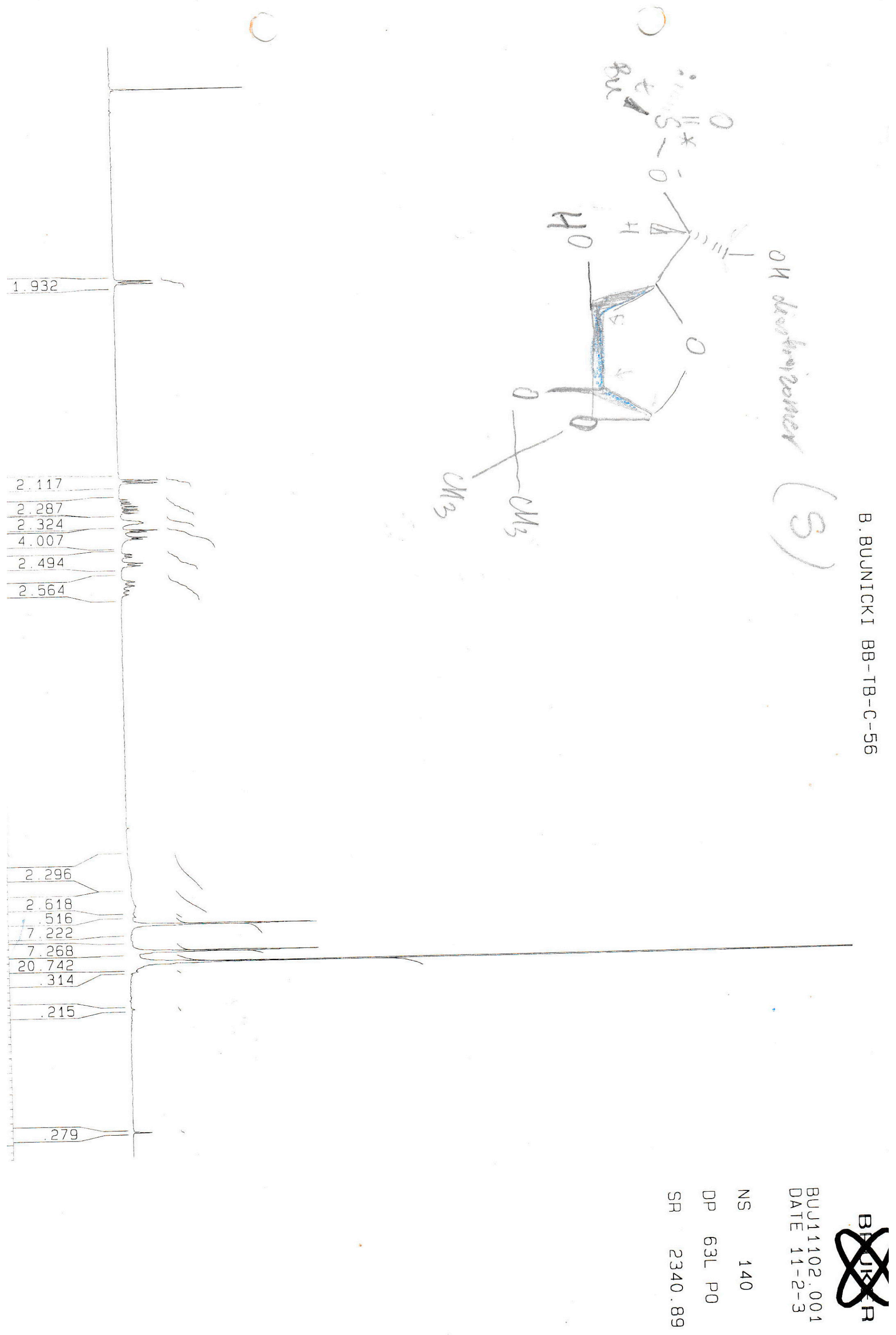

Figure 2S. ^{1}H NMR Spectrum of (*S*)-1,2-*O*-isopropylidene-(5-*O*-α-D-glucofuranosyl) *t*-butanesulfinate (*S*)-**10**

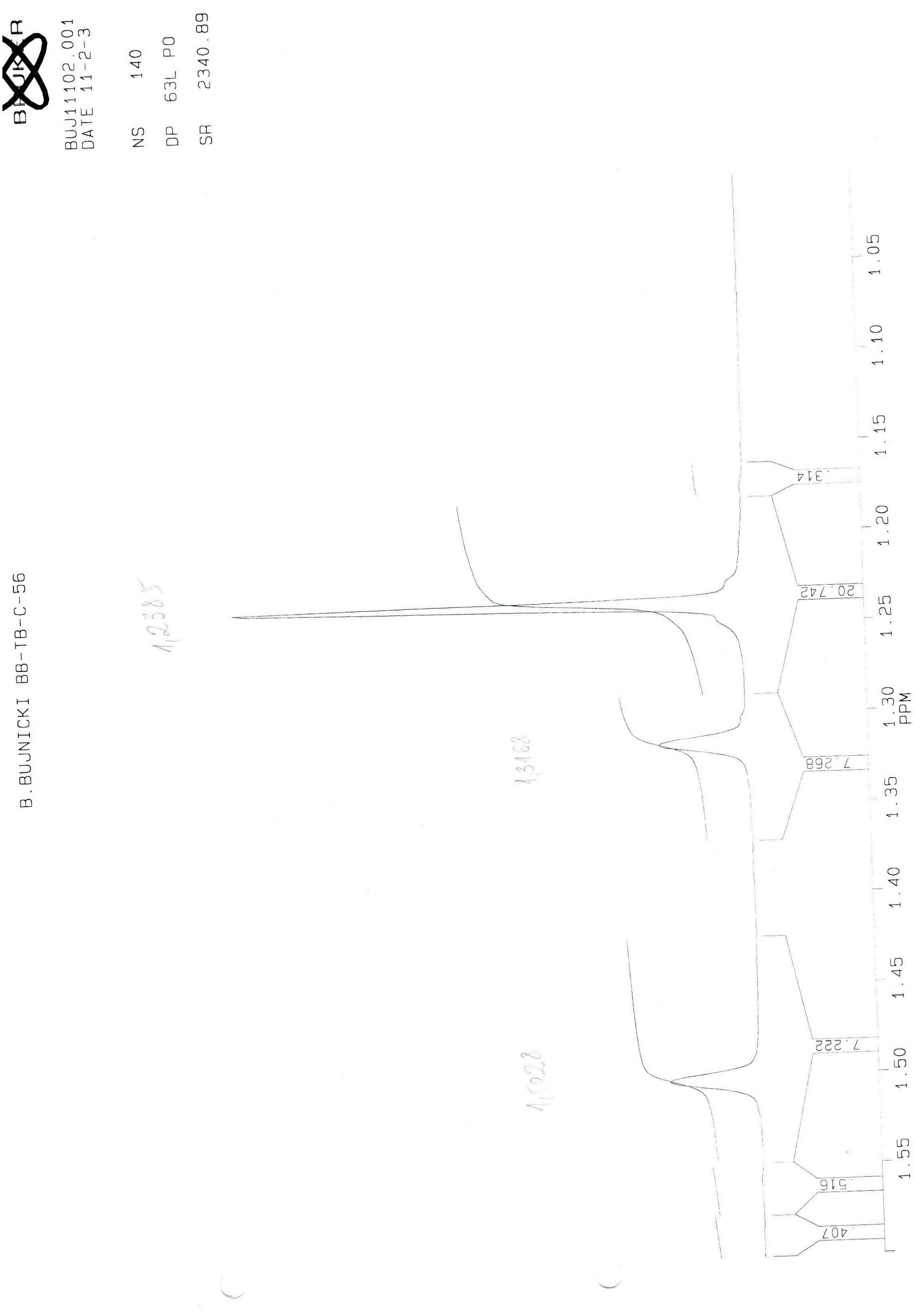

Figure 3S. ^{1}H NMR Spectrum of (*S*)-1,2-*O*-isopropylidene-(5-*O*-α-D-glucofuranosyl) *t*-butanesulfinate (*S*)-**10** (Range extension 1.0-1.6 ppm)

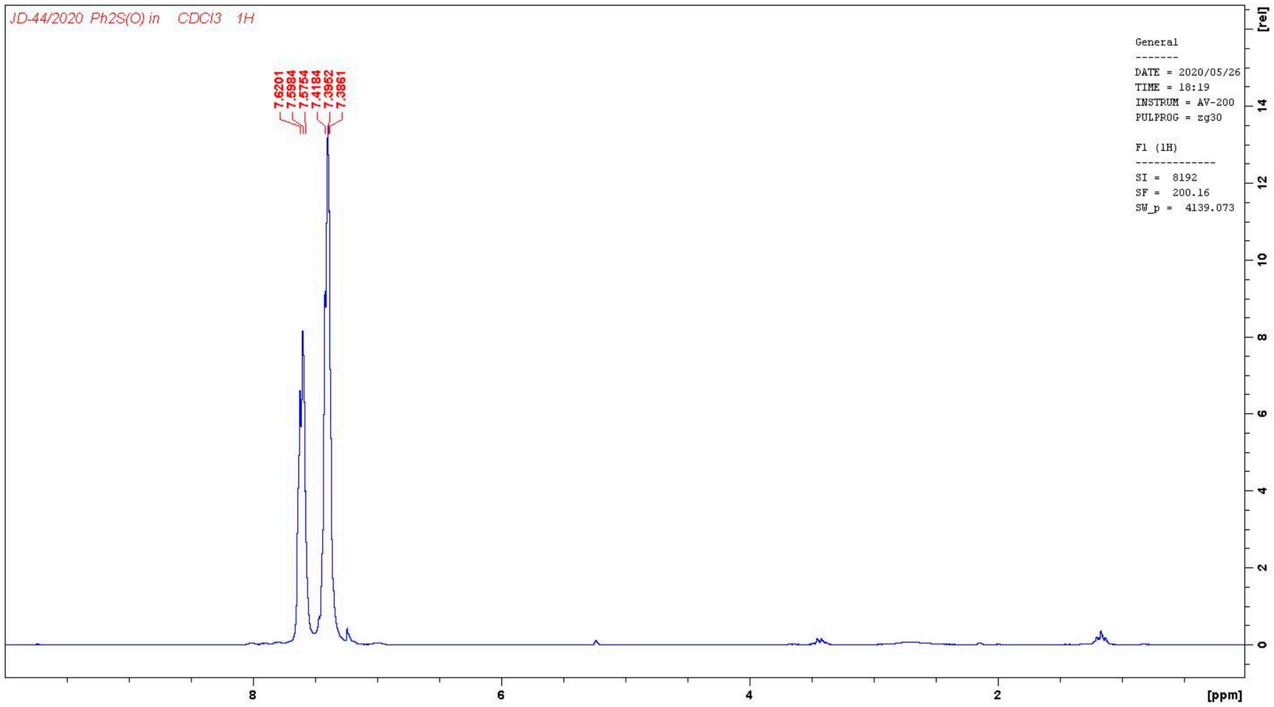

Figure 4S. ^{1}H NMR Spectrum of diphenyl sulfoxide

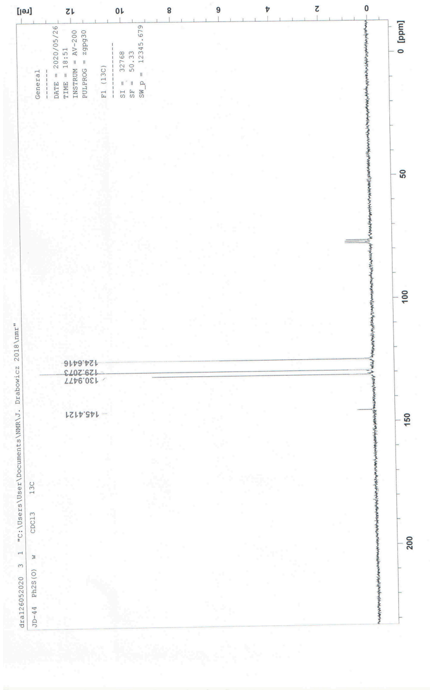

Figure 5S. ^{13}C NMR Spectrum of diphenyl sulfoxide

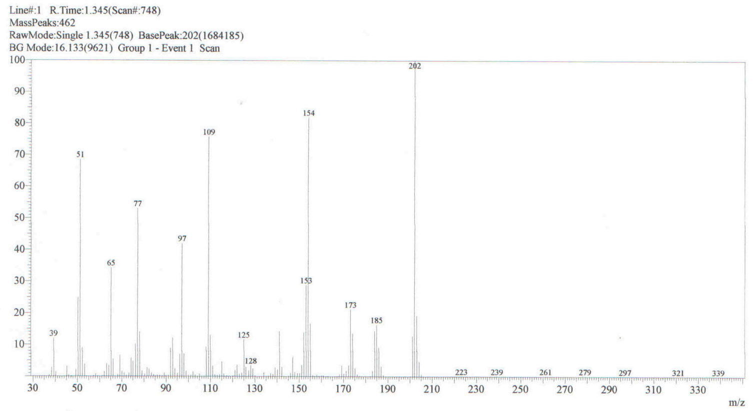

Figure 6. Mass Spectrum of diphenyl sulfoxide

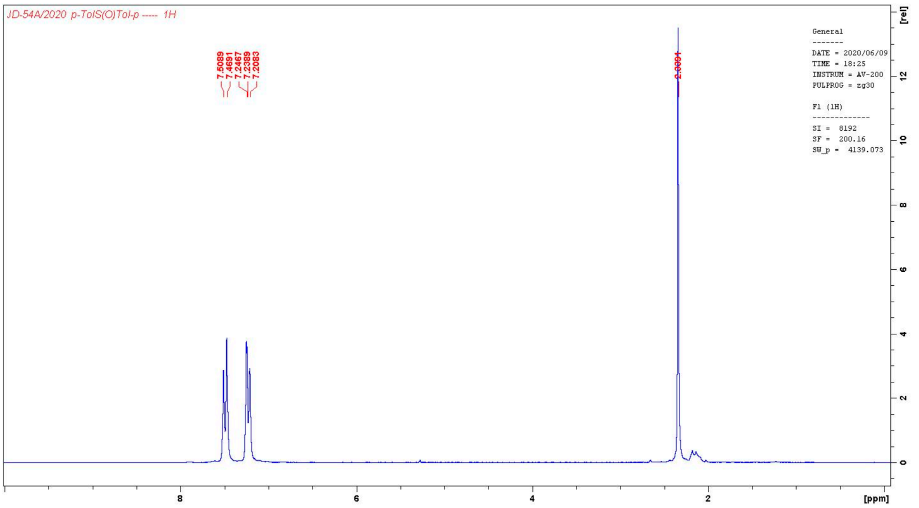

Figure 7S. ^{1}H NMR Spectrum of di-p-tolylsulfoxide

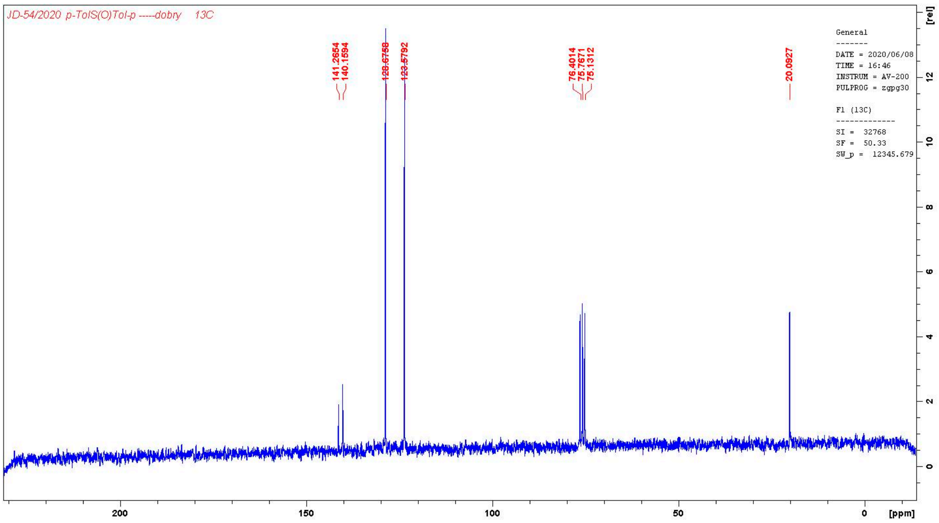

Figure 8S. ^{13}C NMR Spectrum of di-p-tolylsulfoxide

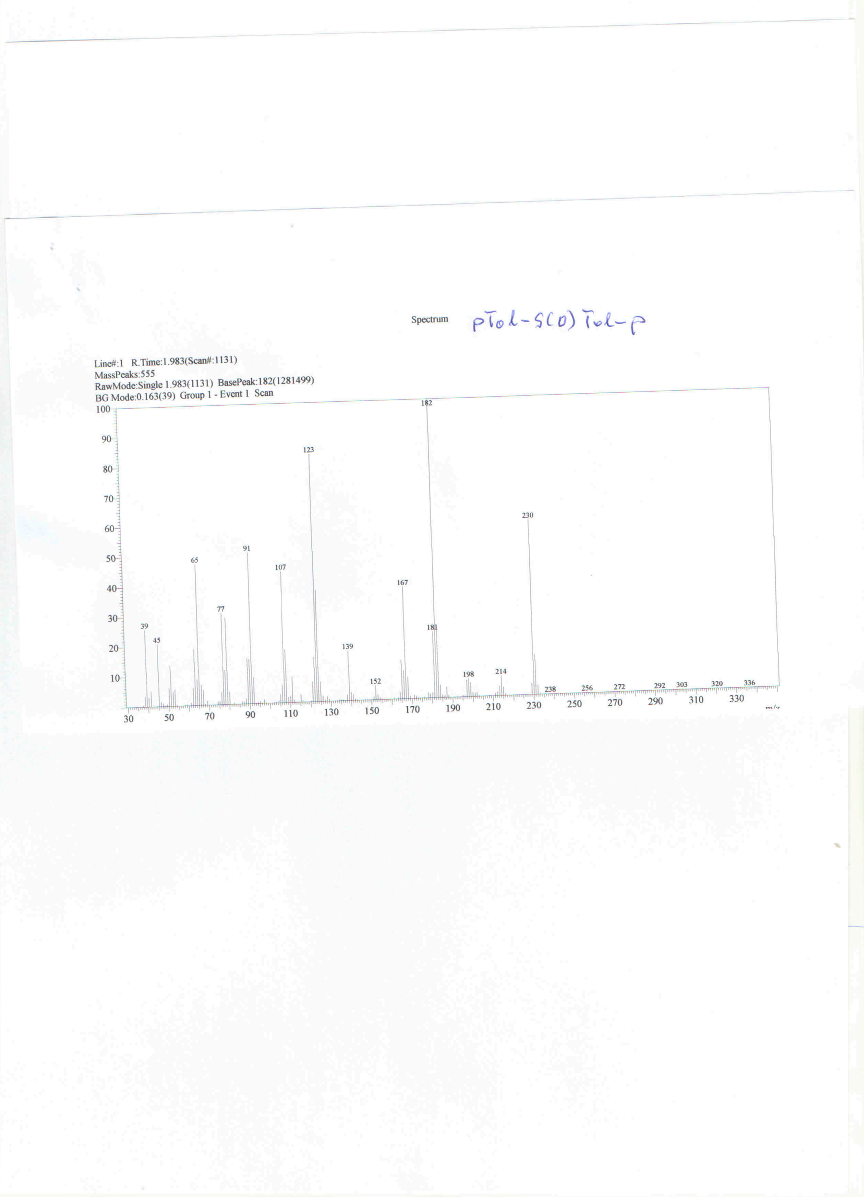

Figure 9S. Mass Spectrum of di-p-tolylsulfoxide
